# Systems thinking in practice: the current status of the six WHO building blocks for health system strengthening in three BHOMA intervention districts of Zambia: a baseline qualitative study

**DOI:** 10.1186/1472-6963-13-291

**Published:** 2013-08-01

**Authors:** Wilbroad Mutale, Virginia Bond, Margaret Tembo Mwanamwenge, Susan Mlewa, Dina Balabanova, Neil Spicer, Helen Ayles

**Affiliations:** 1 Department of Community Medicine, University of Zambia School of Medicine, Lusaka, Zambia; 2 Clinical Research Department, Faculty of Infectious and Tropical Diseases, London School of Hygiene and Tropical Medicine, Keppel Street, London WC1E 7HT, UK; 3 ZAMBART Project, Ridgeway Campus, University of Zambia, Nationalist Road, Lusaka, Zambia; 4 Department of Global Health and Development, Faculty of Public Health and Policy, London School of Hygiene and Tropical Medicine, 15-17 Tavistock Place, London WC1H 9SH, UK

## Abstract

**Background:**

The primary bottleneck to achieving the MDGs in low-income countries is health systems that are too fragile to deliver the volume and quality of services to those in need. Strong and effective health systems are increasingly considered a prerequisite to reducing the disease burden and to achieving the health MDGs. Zambia is one of the countries that are lagging behind in achieving millennium development targets. Several barriers have been identified as hindering the progress towards health related millennium development goals. Designing an intervention that addresses these barriers was crucial and so the Better Health Outcomes through Mentorship (BHOMA) project was designed to address the challenges in the Zambia’s MOH using a system wide approach. We applied systems thinking approach to describe the baseline status of the Six WHO building blocks for health system strengthening.

**Methods:**

A qualitative study was conducted looking at the status of the Six WHO building blocks for health systems strengthening in three BHOMA districts. We conducted Focus group discussions with community members and In-depth Interviews with key informants. Data was analyzed using Nvivo version 9.

**Results:**

The study showed that building block specific weaknesses had cross cutting effect in other health system building blocks which is an essential element of systems thinking. Challenges noted in service delivery were linked to human resources, medical supplies, information flow, governance and finance building blocks either directly or indirectly. Several barriers were identified as hindering access to health services by the local communities. These included supply side barriers: Shortage of qualified health workers, bad staff attitude, poor relationships between community and health staff, long waiting time, confidentiality and the gender of health workers. Demand side barriers: Long distance to health facility, cost of transport and cultural practices. Participating communities seemed to lack the capacity to hold health workers accountable for the drugs and services.

**Conclusion:**

The study has shown that building block specific weaknesses had cross cutting effect in other health system building blocks. These linkages emphasised the need to use system wide approaches in assessing the performance of health system strengthening interventions.

## Background

In the year 2000, the United Nations millennium declaration was signed by 189 member countries. These were later translated into eight millennium development goals (MDGs) which were to form a basis for development and poverty eradication throughout the world. Out of the eight MDGs, three are directly related to improvement of health [1,2].

The drive to produce results for the MDGs has led many stakeholders to focus on their disease priority first. However, recent evidence has lead to concerns that many member countries especially in poorer nations will be unable to meet the MDG targets by the year 2015 [1,3]. Experience to date, suggests that if health systems are lacking capabilities in key areas such as the health workforce, drug supply, health financing, and information systems, they may not be able to respond adequately even if there was an increased in funding and technical support. Furthermore, there is concern that already weak systems may be further compromised by over-concentrating resources in specific programmes, leaving many other areas further under-resourced [3,4]. It has now been recognised that a primary bottleneck to achieving the MDGs in low-income countries is health systems that are too fragile and fragmented to deliver the volume and quality of services to those in need [3,4]. Strong and effective health systems are increasingly considered a prerequisite to reducing the disease burden and to achieving the health MDGs, rather than the outcome of increased investments in disease control. As a consequence, health systems strengthening (HSS) has risen to the top of the health development agenda.

In order to justify continued investments in health systems, there is need to generate evidence that such investment lead to improvement in health [5]. Hence, the design and evaluation of health system strengthening interventions need to be rigorous and robust [6]. In this regard, WHO has proposed a framework of health system building blocks that describes six sub-systems of overall health system architecture. The building block approach could help in identifying bottlenecks in the health system and guide efforts in resource allocation and performance evaluation [7]. Anticipating how an intervention might flow through, react with, and impinge on these sub-systems is crucial and forms the opportunity to apply systems thinking in a constructive way in health system strengthening [8,9].

In recent times, public health researchers and practitioners have been turning to systems thinking to tackle complex health problems and risk factors. Recent projects have used systems thinking to address specific public health problems like tobacco consumption, obesity and tuberculosis [10-12]. In its recent report, WHO has noted that systems thinking has huge and untapped potential in addressing broader health systems problems [8]. It has been argued that application of systems thinking could help in identifying leverage points in a complex health system and could be valuable in guiding the design and evaluation of public health interventions [13,14].

Zambia is one of the countries that are lagging behind in achieving millennium development targets. Several barriers have been identified as hindering the progress towards health related millennium development goals. These include socio-cultural practices, poor referral systems, limited health infrastructure and lack of qualified health human resources [15]. These barriers limit access to health services especially in rural areas. Designing an intervention that addresses these barriers was crucial. The Better Health Outcomes through Mentoring and Assessment (BHOMA) project was born with the current challenges in the Zambia’s MOH in mind and the need to provide a system wide solution rather than disease specific. The BHOMA project was designed to work at district, community and health facility level in the target districts. The full methodology of the BHOMA study is described elsewhere [16].In this paper, we applied systems thinking approach to describe the baseline status of the six WHO building blocks. The main objective was to provide a baseline qualitative analysis of the status of the health systems building blocks before the implementation of the BHOMA intervention in the target districts. This qualitative paper complements baseline quantitative results reported elsewhere [17].

## Methods

We used qualitative ethnographic methods to analyse the status of the Six WHO building blocks in three BHOMA districts using systems thinking approach. The three districts were purposefully sampled to act as pilot districts for an innovative health system intervention with the aim of learning and rolling out the intervention to others districts. The other selection criteria were that these must be rural districts and have similar health system challenges to other rural districts in Zambia. The study was conducted between January and March 2011.We conducted key informant interviews and focus group discussions.

### Target groups

Focus group discussions (FGDs)

Men aged between 18–35 years

Women aged between 18–35 years, with at least one child or more

Key Informant Interviews

Facility level

The In-charge at health facility

Neighbourhood health committee Chairperson or representative

Pharmacist

District Level

Clinical care specialist

District Director of health

### Sampling and size

A total of three districts and nine health facilities were included in the study. Three health facilities were selected in each district. The selection criteria were that in each district one rural, one semirural and one urban health facility was to be included. Where there was more than one eligible health facility one was randomly selected.

At each facility, the health centre in-charge, Chairperson of the Neighbourhood health committee (NHC) and a pharmacist were interviewed.

Around the catchment area of each health facility, two Focus Group Discussions (FGDs) were held with men and women. In total 30 key informant and 18 FGDs were conducted.

### Selection for FGD participants

Community groups were organized with the help of local leaders and community health representatives who helped in informing community members about the dates and time of the interview. Attention was paid to the group heterogeneous characteristics, i.e. different occupations, social networks, educational status. Men and women were interviewed separately.

All group discussions were held away from the health facility to avoid influence from the health workers .All interviews were recorded and later transcribed by trained research assistants familiar with qualitative methods. The transcribed material was validated by the team leader.

### Data collection

Three different interview guides were used for data collection. Two separate key informant interview guides targeting health workers and community representatives and one Focus group discussions guide for collecting information from community members were developed. The themes and questions were based on literature and reported challenges in the Zambian health system. The questions were pre-tested in pilot health facilities within the BHOMA intervention and adapted to reflect the Zambian health care settings. Focus group discussion guides were translated into local languages spoken in study sites. Key informant interviews were conducted in English except those for community representatives. Questions covered the six building blocks for health system from both demand and supply side:

•Service delivery: Access and barriers to health services.

•Health human resources: Availability, gender and attitude of health workers.

•Medical supplies: Availability and stock out of selected medical supplies.

•Governance: Accountability and community participation.

•Health information: Information flow from health facility to the community.

•Finance: User fees and indirect payments,

Data was collected by the research team comprising the main researcher and three research assistants trained in qualitative methods.

### Data analysis

Data was transcribed by five research assistants trained in qualitative methods. All scripts were checked and validated by the main researcher. Transcripts were cleaned and exported to Nvivo 9 for analysis. Coding was done by the main researcher and checked by the second researcher experienced with qualitative methods. Data coding followed pre-determined themes based on health system building blocks. These formed the basis for broader themes which were further subcategorised to increase the explanation ability of the data.

### Ethical consideration

The study was approved by the University of Zambia Biomedical Ethics Committee and London School of Hygiene and Tropical Medicine. All participants were informed about the study and signed a consent form before being enrolled in the study. Confidentiality was maintained throughout data collection, analysis and publication.

## Results

### Health service delivery building block

#### barriers to accessing health services

Several barriers were identified as hindering access to health services by the local communities. These included supply side barriers: Shortage of qualified health workers, bad staff attitude, poor relationships between community and health staff, long waiting time, confidentiality and the gender of health workers. Demand side barriers: Long distance to health facility, cost of transport and cultural practices.

### Staffing, attitude and waiting time

The staffing levels at health facilities appeared to have a bearing on the patient/provider relationship. It appeared that it was not possible to improve the relationship between the community and the health facility by simply increasing the number of health workers disregarding the issues of behaviour and attitude of health workers. Most members of the community were discouraged from seeking medical attention if the health workers were rude and uncaring. Some community members only came to seek services if the right health workers were on duty. While most health workers blamed the bad relationship between the community and health workers on fewer numbers of health workers, the community members felt that it was not enough to have more health workers. They insisted that the health workers must be caring and have a positive attitude towards work. Waiting time before being seen by a health worker was one indicator of not only the low number of health workers but also a reflection of bad working practices and attitude by health workers. The long waiting hours were a recipe for poor relationship and this was self-reinforcing:

“Staffing should be improved at the clinic. If the clinic has adequate staff when patients come they will spend less time at the clinic.”

*Male FGD participant, Chongwe*

### Community attitude

Community members have a duty to help health workers to perform their duty without risking their lives. Therefore, the issue of improving relationships at health centres has both demand and supply side. Findings from our study showed that the community does not seem to see their responsibility to be crucial in improving relationships with health workers. Community members expected health workers to improve their attitude and not the community needing to change to accommodate health workers. Sometimes the community delayed in seeking medical help until the case was very serious. This was seen as bad community practice that needed to change. However, the community blamed the delays on health workers who they said were unwilling to attend to none serious cases, so community members had no choice but to wait until the illness was very serious in order to draw attention from health workers.

“You see, other people stay far away from the clinic and have no money for transport.

This makes them to delay in seeking health care until the illness becomes very serious.”

*Male NHC chairman, Luangwa*

### Inequalities in access to health services

Access to health services is vital for all age groups and gender. In our study area, services seemed to favour women and children. Participants reported that men were usually bottom on the list when it came to receiving help from health services from the health facility. This was reflected in the following quote:

“In most cases when children have got problems they are given medication including injections.

Adults are usually told that drugs are out of stock so they have to buy on their own.”

*Male FDG participant, Kafue*

#### Confidentiality

Stigma remained a challenge in accessing HIV and sexually transmitted diseases services. Many clients feared that health workers could breach confidentiality if they were told about such sensitive matters. In contrast, NHC members believed that health workers maintained confidentiality at all times. One such service negatively affected by stigma was couple counselling for HIV which has remained low.

“Sometimes patients are not free to talk to health workers, for example they may have an STD but it may be difficult for them to explain to the health workers until the condition becomes very bad. In some cases, patients have died at home that is when people discover that they were suffering from this and that disease.”

*Male, FDG participant, Luangwa*

#### Distance and transport costs

Long distance from health facilities and cost associated with transporting patients to local health facilities and referral centres were the major demand side barriers to accessing health services on time. There was limited access to ambulance services in most rural health facilities and in some cases patients referred to hospital were asked to arrange their own transport. This resulted in some referred patients staying and dying at home because they could not afford transport costs. In fact services rated very poorly in most health centres were those needing referral to other institutions to complete the management of illness.

*“Sometimes when the patient is very sick they are unable to sit on a bicycle and are unable to walk so you find that it would even take time for them to come and reach the health services.”****HC, in-charge, Kafue***

“For T.B, we still have problems in the sense that for us to know that a person has T.B, when they get sputum they have to take it to a bigger hospital for laboratory testing after testing if they find T.B that is when they come here to receive T.B drugs.”

*HC in-charge, Chongwe*

### Health human resources building block

#### Shortage of health human resource

The density of qualified human resources has been found to be important in improving the quality of health services. In the absence of trained health workers service delivery could be severely compromised [18,19]. In our study we found that there was a general shortage of qualified human resources such that patients were sometimes attended to by cleaners and other untrained staff.

“The health workers are not enough sometimes. When you have a patient you take him to the clinic and if the nurse is not around, then you are attended to by the cleaner who is not trained to do that job so we need more nurses.”

*Male FDG participant, Luangwa*

#### Task shifting: are the unpaid daily employees (CDEs) the answer?

In recent times, there has been an increased emphasis on task shifting as a solution to the problem of staff shortages for qualified health workers. This has taken different forms in many countries with some volunteers or paid lay workers taking up some roles originally done by qualified health workers [20-23]. In Zambia, a cadre known as Classified Daily Employees (CDEs) are helping health workers to perform some tasks which they have never been trained to do. Though some of them have now been put on government payroll, most of them work on voluntary basis and perform tasks ranging from patient screening to prescribing and despising drugs.

The findings showed that, although CDEs were seemingly willing to help at the health centre, they appeared to have deep seated bitterness for not being paid despite their contribution. Though most of them accepted that their work was supposed to be voluntary, after working for some time, they generally felt entitled to remuneration and indirectly showed signs of demanding payments for their work. They contended that they were overwhelmed with responsibilities and required to work awkward hours just like trained health workers yet without pay. There was a feeling of abuse and helplessness among many CDEs.

“According to me we still have so many problems because even as I am here we are not paid. You see because of the job I do here, I am unable to do other jobs which can give me money, but instead I help the people (staff) who are paid by the government so that is a big problem for me.”

*Male CDE, Luangwa*

#### Suggested motivation for CDEs

Although some CDEs are currently working on voluntary basis, they were keen to receive incentives as a motivation as well as a sign that their work was being appreciated. Though money was seen as important, other forms of motivation highlighted during the study were less complicated than initially thought. These included positive complements, simple certification, training opportunities and being given priority when new research projects are being implemented in the area.

#### Gender and age of health workers and health facility delivery

The gender of health workers can positively or negative affect access to health services. The type of health services likely to be affected may differ by area of residence and cultural beliefs. In our study male nurses were seen as a hindrance to health facility deliveries as many women expressed reservations to being attended to by male nurses during labour. This could be a cultural issue which is more pronounced in rural areas than urban. For example in one rural health facility in Kafue district, there was only a male nurse attending to patients including pregnant women. He acknowledged that some women were not happy to give birth at the health centre because it was manned by a male health worker and so they preferred to give birth at home or other facilities were female workers were present. This finding was confirmed through interviews with women and men in the community.

*“You know we only have a male nurse here, we need a female nurse. Because of this problem other women give birth from home or go to nearby hospital.”****Female FDG participant, Chongwe***

Younger health workers discouraged older clients from seeking health care as explained by one respondent:

“Some people fail to come to the clinic because of the age of the health workers for instance some are young as a result those who are older than them fail to come to the clinic.”

*Male FDG participant, Chongwe*

### Medical and drug supplies building block

#### Managers’ and community perception of drug availability

The health facility in-charges’ perception of drug availability was interestingly different from the community members who felt that the drugs were not always available. Most health facilities experienced shortages of essential drugs. This was attributed to irregular supplies of drugs by the government.

Interestingly, whether drugs were in stock or not revealed important insights that must be considered when planning for drugs and other medical supplies.

Some drugs were in stock because there were few cases to be treated not because there was a good supply. Other drugs run out because there was higher demand compared to supply. Some health facilities had high population but the drug supplied seemed to be fixed. This was reported in bigger health facilities located in peri urban areas as expressed by one respondent:

*“The population is big and the drugs the government send us are not enough.”****Female FDG participant, Chongwe***

Some drugs run out of stock because they were more used than others. In some cases community demanded to be given certain drugs as they felt these were most helpful. For example, there seemed to be a belief among most community members that flagyl (an antibiotic) was good medicine for diarrheoa and if not given some members of the community felt betrayed. In such instances, some health workers felt obliged to give such medication even when they knew that would not change the course of the illness. This was done for the sake of maintaining confidence and trust in the health services being provided at health facility.

When some medicines run of stock, patients were given prescriptions to buy from private pharmacies. Most clients were not happy to be given prescriptions as they had no money to buy drugs from private pharmacies.

“Sometimes when you go to the clinic they just give you a prescription for you to go and buy medicine.If we can’t afford to buy books, how can we afford to buy medicines from private pharmacies?”

*Female FDG participant, Luangwa*

### Governance at health facility level

Health system governance has many facets. Some elements of governance include transparency, accountability and community participation [24]. We collected information about some elements of governance from both facility managers and community perspectives.

#### Community participation in health services

There were gender differences in community participation in health service provision. Male community members were more likely to participate in health facility initiatives and were well informed about services available at the health facilities and took part in the activities of the health facility. In contrast, most female participants were not aware of the activities that were going on at the clinic. However, when asked about who owned the health services at health facility, most respondents including women said that the health services were owned by the community despite their low participation.

#### Accountability for the resources

It is crucial that members of the community provide checks to health workers to ensure equitable access to health service. The capacity for community to hold health workers accountable vary from community to community and area of residence (rural vs. urban) [25]. In Zambia the Ministry of health has recommended formation of neighbourhood health committees as part of the governance structure for a health facility. These are expected to act as representatives and eyes for the community [26]. We explored the extent to which the communities or their representatives held the health workers accountable for resources especially drugs.

The results showed that, community members including members of NHC assumed that the nurses and clinical officers accounted for the drugs and did not actively ensure that this was done and appeared quite ignorant of the process of accounting for the available drugs and medicines.

The community seemed to be incapable of holding health workers accountable for the drugs and services as in most cases they didn’t know how the health workers did their work and so could not ask intelligent questions as highlighted by one NHC committee member:

“The workers at the clinic are the ones who see to it that the medicines are given to every patient us from the community we don’t know.”

*NHC chairman, Luangwa*

### Finance building block: indirect payments

The Zambian government has abolished user fees in rural areas to protect patients and their families from catastrophic health expenditures [27]. This policy has been adopted and is said to be working in most health centres throughout Zambia. In this study we wanted to confirm whether health facilities were indeed not charging patients for services received. The result showed that although most of the selected health facilities indicated that they did not charge user fees to patients or clients, in reality there seemed to be indirect payments through forcing clients to buy books from health facilities or shops. Those without books were not being attended to by health workers at health facilities. This was seen as a form of payment by most community members who felt discouraged from seeking medical attention even when they were very sick because they could not afford to by a note book. One respondent said:

*“We don’t pay user fees, it is for free. But we are told to buy books from the clinic once we buy from somewhere else they refuse to write in them.”****Female FDG participant, Chongwe***

User fees were officially requested for patients crossing from Mozambique into Zambia seeking medical attention in Luangwa district. This was not the case in Chiawa (Kafue) were Zimbabweans seeking health services in Zambia did not pay any form of user fees.

### Health information

Information flow from the health centre to the community about new services was very inconsistent and appeared to depend on community volunteers. While most health workers assumed that the NHC communicated information to the community, there was no way of verifying this. Most community members denied being aware of new services or initiatives that were being implemented by the health facility. Interesting health information flow appeared to favour males who acquired information through their local social networks more than female respondents.

“The villagers do not know or are not aware of all the programmes offered by the clinic as some of them in most cases have to ask if certain services are offered by the clinic.”

*Female CDE, Luangwa*

## Discussion

The study has shown that building block specific weaknesses had cross cutting effect in other health system building blocks which is an essential element of systems thinking [28]. These linkages emphasis the need to use system wide approaches in assessing the performance of health system interventions [8]. It was clear that challenges noted in service delivery were linked to human resources, medical supplies, information flow, governance and finance building blocks either directly or indirectly.

Service delivery was directly affected by availability of trained health workers. In addition, the attitude and gender of health workers were other key human resource attributes that affected access to health services.

While the concentration of health workers was important, their behaviour and attitude toward patients was even more important to the community. Bad health worker attitude discouraged some people from going to health facility and was cited as a reason for long waiting time at health centres rather than the lack of human resources. Other studies have reported similar concerns about the attitude of health workers and how it has an influence on service utilisation [29].

The gender of health workers was an important consideration when it came to health facility deliveries. Most female participants were reluctant to deliver at the health centre if the only health worker available was male. In such cases, clients preferred to deliver at home or being assisted by traditional birth attendant. The refusal by most women and their partners to be attended to by a male health worker during labour was common in rural health facilities where it was seen to be culturally inappropriate to be attended by the opposite sex during labour. This finding has implication when it comes to attainment of millennium development goals on maternal and child health [1,30].

Another factor noted as a hindrance to accessing HIV and STI services was perceived lack of confidentiality on the part of health workers. Most community members feared that health workers would leak information about their HIV or STI status if they went to the nearby health facility. This resulted in delays in seeking medical attention with associated complications. The fear of breaching confidentiality has been reported in Zambia even among health workers when they come to seek HIV services [31]. It is important that health workers are trusted by community to keep confidential information. If patients are assured of confidentiality, they are more likely to seek medical attention early at the nearest health centre [32].

In few places, there were no qualified health workers and such health facilities were being manned by unqualified personnel known as Classified Daily Employees (CDEs). Similar findings have been reported in other districts within Zambia [33]. These usually worked on voluntary basis. The study findings showed that they had no formal training or evaluation. With most health facilities having only one trained health worker, in the absence of trained health, the responsibility fell on CDEs. This puts the patient’s lives at risk and severely compromised quality of service delivery [22]. While most of them were doing their best to help on voluntary basis, the indications were that this was not sustainable. Most CDE wanted to be trained and to receive allowances and other incentives which were not currently available. While task shifing has been noted to be successful elsewhere, its success has depended on training of lay workers to do specific tasks and not necessarily managing patients on their own as this responsibility falls on qualified health workers [20,34,35].

There was a general feeling by the community that essential medical supplies were usually out of stock. Rapid diagnostic tests for malaria (RDT) and antibiotics were said to be out of stock most of the time. This had negatively affected trust in the health system as most participants felt cheated when they were only given prescription to buy medications which were not in stock. The reason for stock out was that supply of medicines was fixed while the demand had kept increasing. It was not possible to know whether health workers misused the medical supplies as the community and its representatives lacked capacity to hold health workers accountable for medical supplies. They simply trusted that health workers were doing a good job.

Access to health services had been declared free in Zambia following removal user fees [27]. This was reported to be the case in all health centres except a few places in Luangwa where user fees were charged to foreigners seeking health care in Zambia. Nonetheless, there was a requirement that patient provided their own note book for medical notes. This condition was seen by many as a form of payment and was discouraging some people from seeking medical attention.

Another barrier to accessing health services was long distance to health facilities which translated into high transport for patients and their families [36]. The lack of ambulance compounded the problem as most clients were required to facilitate and pay for their own transport and lodging to referral centres. It was therefore not surprising to find that most services needing referral were among the worst performing.

## Conclusion

In summary, there were close linkages between service delivery and other health systems building blocks. Challenges affecting particular building blocks seemed to have ramification in other building blocks directly or indirectly. For example, the attitude, behaviour, gender and age of health workers seemed to have an effect on trust and demand for health services. It is therefore essential to apply system wide approaches when evaluating health systems due to close linkages that exist between sub-systems. It was clear that the success or failure reported in one building block accounted for success or failure reported in other building blocks.

## Competing interests

The authors declare that they have no competing interests.

## Authors’ contributions

WM: Participated in study design, data collection and analyzed the data and drafted the manuscript. VB: Participated in study design and provided critical review of the manuscript. MTM: Was involved in designing the BHOMA project and reviewed the manuscript. SM: Participated in data collection and analysis and reviewed the manuscript. DB: Provided critical review of the manuscript from a health systems perspective. NS: Provided critical review of the manuscript from a health policy perspective. HA: Designed the BHOMA project and provided critical review of the manuscript. All authors read and approved the final manuscript.

## Pre-publication history

The pre-publication history for this paper can be accessed here:

http://www.biomedcentral.com/1472-6963/13/291/prepub
